# The Microbe Directory: a centralized database for biological interpretation of microbiome data

**DOI:** 10.1093/database/baaf060

**Published:** 2025-09-24

**Authors:** Maria A Sierra, Krista Ryon, Mohith R Arikatla, Radwa Elshafey, Hardik Bhaskar, Jacqueline Proszynski, Chandrima Bhattacharya, Heba Shaaban, David C Danko, Pradeep Ambrose, Sarah A Spaulding, Maria Mercedes Zambrano, The Microbe Directory Consortium, Christopher E Mason

**Affiliations:** Tri–Institutional Computational Biology and Medicine Program, Weill Cornell Medicine, 445 East 69th Street, Room 205, New York, NY 10021, United States; Department of Physiology and Biophysics, Weill Cornell Medicine, 1300 York Avenue, Rm E509, New York, NY 10065, United States; Weill Cornell Graduate School of Medical Sciences, 1300 York Ave. Box 65, New York, NY 10065, United States; Hunter College, City University of New York, 695 Park Avenue, New York, NY 10065, United States; Hunter College, City University of New York, 695 Park Avenue, New York, NY 10065, United States; Department of Physiology and Biophysics, Weill Cornell Medicine, 1300 York Avenue, Rm E509, New York, NY 10065, United States; Tri–Institutional Computational Biology and Medicine Program, Weill Cornell Medicine, 445 East 69th Street, Room 205, New York, NY 10021, United States; Joan & Sanford I. Weill Medical College of Cornell University, 1300 York Ave. New York, NY 10065, United States; Tri–Institutional Computational Biology and Medicine Program, Weill Cornell Medicine, 445 East 69th Street, Room 205, New York, NY 10021, United States; Department of Physiology and Biophysics, Weill Cornell Medicine, 1300 York Avenue, Rm E509, New York, NY 10065, United States; Institute of Arctic and Alpine Research, University of Colorado Boulder, 4001 Discovery Dr, Boulder, CO 80303, United States; Molecular Genetics, Corporacion CorpoGen Research Center, Carrera 4 # 20-41, Bogotá, Colombia; Tri–Institutional Computational Biology and Medicine Program, Weill Cornell Medicine, 445 East 69th Street, Room 205, New York, NY 10021, United States; The HRH Prince Alwaleed Bin Talal Bin Abdulaziz Alsaud Institute for Computational Biomedicine, Weill Cornell Medicine, 1305 York Avenue, New York, NY 10065, United States; WorldQuant Initiative for Quantitative Prediction, Weill Cornell Medicine, 1300 York Avenue, New York, NY 10065, United States; The Feil Family Brain and Mind Research Institute, Weill Cornell Medicine, Belfer Research Building 413 E 69th St, New York, NY 10021, United States

## Abstract

The Microbe Directory (TMD) is a centralized database of metadata for microbes from all domains that helps with the biological interpretation of metagenomic data. The database comprises phenotypical and ecological traits of microorganisms, which have been verified by independent manual annotations. This effort has been possible by the help of a community of volunteer students worldwide who were trained in manual curation of microbiology data. To summarize this information, we have built an interactive browser that makes the database accessible to everyone, including non-bioinformaticians. We used the TMD data to analyse microbiome samples from different projects such as MetaSUB, TARA Oceans, Human Microbiome Project, and Sponge Microbiome Project, showcasing the utility of TMD. Furthermore, we compare our microbial annotations with annotations collected by artificial intelligence (AI) and demonstrate that despite the high speed of AI in reviewing and collecting microbial data, annotation requires domain knowledge and therefore manual curation. Collectively, TMD provides a unique source of information that can help to interpret microbiome data and uncover biological associations.

**Database URL**: www.themicrobedirectory.com/

## Introduction

The development and use of databases in science is not a recent phenomenon. In recent decades, databases have become a cornerstone of biological research, largely due to advances in molecular techniques [1], which have enabled access to the hundreds to millions of letters that make up a genome.

The first sequenced genome—a bacteriophage—completed by Fredrick Sanger in 1977 [2], was published in its entirety within the manuscript, as no public storage repositories existed at the time to house the 5386-base pair (bp) genome. As a comparison, if the human genome had instead been printed, it would have required more than 1.5 million paper pages to contain the 3.1 billion bp sequence. The sequencing of large genomes, especially the Human Genome Project (HGP), highlighted the need of a centralized method to store sequence information that could be easily accessed, and shareable among research teams worldwide [3, 4].

Today, the scale of sequencing has expanded dramatically, particularly in the field of metagenomics. To date, more than 32,000 research articles describing metagenomes have been published in PubMed. Most of these studies represent newly sequenced metagenomes—a compilation of genomes that likely represent hundreds or thousands of species. Manual investigation of these complex datasets is time-consuming and may lead to a never-ending data curation process. This represents an immediate challenge for researchers attempting to interpret biological data and generate biological hypotheses from sequencing results.

The Microbe Directory (TMD) database was developed as a necessity for a free, user-friendly, and centralized database of microbial features and ecological characteristics. It is designed to be accessible to students, scientists, and clinicians. TMD is not a sequence repository, nor a taxonomic classification tool. Instead, TMD compiles metadata of microbes from all domains of life to aid with the interpretation of metagenomic outputs. By manual annotation and curation, TMD has compiled information to date on almost 18,000 species of bacteria (5201), archaea (873), algae (4209), fungi (3926), and viruses (3,596). The database provides information on 42 microbial features such as the type of genetic material in viruses and the type of Gram-staining for bacteria, as well as ecological characteristics such as the source of the microbiome (e.g. water or soil) or the ability to withstand extreme conditions (i.e. extremophiles). This represents a significant upgrade from the first two versions of the database [5, 6], with a more comprehensive dataset, extended annotations, and a user-friendly data interface that empowers users to navigate and utilize the database effectively.

Lastly, we underscore the importance of manual annotations in TMD, comparing them with annotations generated by artificial intelligence (AI). Despite the recent strides made in AI, our findings demonstrate the continued necessity for a domain of knowledge and, consequently, manual curation in creating and maintaining databases like TMD.

## Methods

### Volunteers training and data curation

To build the database, manual annotations were performed during the years 2019–22 with the help of almost 400 volunteers. Most of these volunteers were undergraduate students, with some high school and graduate students also participating. The students were recruited primarily through postings on social media and word-of-mouth among students. Volunteers joined the project from various cities in the USA, as well as other countries, including India, Colombia, Ecuador, and Brazil. The selection criteria included only a basic knowledge in microbiology, as the project aimed to provide students with tools and opportunities for remote learning.

All volunteers were trained to read articles, search on repositories, and identify information relevant for the annotation process by Zoom training sessions, video tutorials, and documented guide. This method ensured that all volunteers had an understanding of the research articles, the questions queried in the database, the microbial annotation, and submission process. Each week, the volunteers received a list of 10 microbial species by email and spent on average 8–13 h per week researching the species assigned, and submitting the information queried. Volunteers researched information of microbes from different articles and databases such as PubMed, the American Type Culture Collection (ATCC), MycoBank [5], Global Biodiversity Information Facility (GBIF) [6], eHOMD [7], GOLD [8], BacDive [9], and Diatoms.org [10], along with other openly accessible sources listed in the provided microbiology guides.

To compile information, volunteers submitted their microbial annotations in a customized survey in KoboToolBox [11], Supplementary Fig. 1. Information compiled by each volunteer was later summarized into a single file and cleaned from typos or redundancies using a custom script (see GitHub in the Data availability section).

### Data compilation and processing

A list of all microbial species names, including bacteria, archaea, virus, fungi, and algae, were downloaded from NCBI taxonomy in the following link: https://ftp.ncbi.nlm.nih.gov/pub/taxonomy/ version 2020-07-01 in a taxdump format. The format contains the full taxonomic structure used by NCBI for classifying organisms. It is widely used in bioinformatics for mapping organism names to their taxonomy, navigating the taxonomic hierarchy, map organism names to NCBI taxon IDs, and normalizing organism names across databases [12].

The species list was screened for redundant names using a list of exclusion words for each microbial group using a custom script available in our GitHub (https://github.com/mariaasierra/themicrobedirectory). The complete list of exclusion terms for each microbial group can be found in Supplementary Table 1.

Eukaryotic microorganisms do not differ taxonomically to multicellular organisms. For example, the microalgae *Chlamydomonas* sp. and macroalgae, *Ulva* sp. also known as sea lettuce, are within the same phylum clade Chlorophyta [13]. Therefore, species lists downloaded using the taxdump NCBI format included species of macroorganisms. To compile a list of only microbial eukaryotes, macrofungi and macroalgae were removed from the species list based on a literature review.

To compile the list of microfungi, known genera to comprise only macrofungi (with fruiting body) were removed from the species list retrieved from NCBI taxonomy. All genera of fungi were kept, except *Chlorociboria, Paxina, Helvella, Morchella, Kalaharituber, Peziza, Pseudohelotium, Terfezia, Anthracobia, Isaria, Scutellinia, Rhizina, Phillipsia, Tuber, Choiromyces, Cordyceps, Daldinia, Poronia, Xylaria, Penzigia, Agaricus, Arachnion, Battarrea, Battarreoides, Bovista, Calvatia, Chlamydopus, Chlorophyllum, Coniolepiota, Coprinellus, Coprinopsis, Coprinus, Crucibulum, Disciseda, Gyrophragmium, Langermannia, Lepiota, Leucoagaricus, Lycoperdon, Macrolepiota, Montagnea, Montagnites, Mycenastrum, Parasola, Polyplocium, Psalliota, Secotium, Tulostoma, Xanthagaricus, Amanita, Amanita, Saproamanita, Bolbitius, Conocybe, Galeropsis, Pluteolus, Broomeia, Clavaria, Clavaria, Clavulinopsis, Mucronella, Cortinarius, Locellina, Chondrostereum, Cyphella, Claudopus, Clitopilus, Entoloma, Fistulina, Laccaria, Hygrocybe, Hygrophorus, Anellaria, Panaeolina, Panaeolus, Astrosporina, Crepidotus, Inocybe, Phaeoglabrotricha, Phaeosolenia, Lyophyllum, Podabrella, Termitomyces, Calyptella,Marasmius, Solenia, Cruentomycena, Favolaschia, Mycena, Flagelloscypha, Lachnella, Cyathus, Anthracophyllum, Gymnopus, Marasmiellus, Omphalotus, Phellorinia, Armillaria, Armillariella, Cyptotrama, Oudemansiella, Hymenopellis, Physalacria, Xerula, Pleurotus, Pluteus, Volvariella, Podaxis, Ozonium, Psathyrella, Pterula, Schizophyllum, Sebacina, Agrocybe, Deconica, Flammula, Galera, Gymnopilus, Hebeloma, Hymenogaster, Hypholoma, Kuehneromyces, Leratiomyces, Naucoria, Pholiota, Psilocybe, Stropharia, Tubaria, Amparoina, Cellypha, Clitocybe, Collybia, Lepista, Macrocybe, Melanoleuca, Omphalia, Tricholoma, Tricholomopsis, Tricholosporum, Trogia, Auricularia, Eichleriella, Exidia, Heterochaete, Aporpium, Aureoboletus, Boletus, Buchwaldoboletus, Chalciporus, Imleria, Leccinum, Octaviania, Xerocomellus, Phlebopus, Coniophora, Gyrodontium, Gyroporus, Melanogaster, Paxillus, Rhizopogon, Pisolithus, Scleroderma, Serpula, Suillus, Cantharellus, Pellicularia, Clavulina, Corticium, Cytidia, Dendrothele, Laetiporus, Tretopileus, Geasteropsis, Geastrum, Geastrum, Anthurus, Aseroe, Blumenavia, Clathrella, Clathrus, Ileodictyon, Itajahya, Jaczewskia, Kalchbrennera, Kalchbrennera, Mutinus, Phallus, Daedalea, Fomitopsis, Gloeocystidium, Phaeolus, Rhodofomitopsis, Amauroderma, Ganoderma, Gloeophyllum, Ramaria, Clavariadelphus, Coltricia, Fomitoparia, Fuscoporia, Hydnum, Hymenochaete, Phellinus, Polystictus, Trichaptum, Cotylidia, Grandinia, Heterochaete, Oxyporus, Crustodontia, Acia, Aegerita, Bjerkandera, Cymatoderma, Gloeoporus, Irpex, Laschia, Merulius, Mycoleptodon, Odontia, Phlebia, Podoscypha, Pseudolagarobasidium, Abortiporus, Coriolopsis, Coriolus, Daedaleopsis, Favolus, Fomes, Funalia, Grammothele, Heliocybe, Hexagonia, Lentinus, Lenzites, Lenzites, Lenzites, Lignosus, Microporus, Nigroporus, Neolentinus, Panus, Perenniporia, Picipes, Phellinus, Polyporus, Pycnoporus, Trametes, Lentinellus, Dentipellicula, Laxitextum, Asterostroma, Dichostereum, Lachnocladium, Peniophora, Lactarius, Lactifluus, Russula, Aleurodiscus, Stereum, Hypochnus, Thelephora, Arrhytidia, Calocera, Dacrymyces, Dacryopinax, Femsjonia, Naematoloma, Tremella, Phaeotremella, Sirobasidium, Pilobolus, Echinostelium, Cribraria, Dictydiaethalium, Licea, Lycogala, Reticularia, Tubifera, Diachea, Diderma, Didymium, Mucilago, Badhamia, Badhamiopsis, Craterium, Fuligo, Leocarpus, Physarella, Physarum, Willkommlangea, Amaurochaete, Comatricha, Enerthenema, Lamproderma, Stemonaria, Stemonitis, Stemonitopsis, Calomyxa, Arcyria, Hemitrichia, Metatrichia, Oligonema, Perichaena, Trichia, Ceratiomyxa*, and *C eratium*.

To compile only microalgae, all taxa from the divisions Chlorophyta, Streptophyta, Rhodophyta, and Glaucophyta were kept, except Leiosporocerotopsida, Sphagnopsida, Ginkgoopsida, Andreaeobryopsida, Takakiopsida, Gnetopsida, Andreaeopsida, Haplomitriopsida, Pinopsida, Bryopsida, Jungermanniopsida, Magnoliopsida, Charophyceae, Oedipodiopsida, Marchantiopsida, Lycopodiopsida, Polytrichopsida, Polypodiopsida, Anthocerotopsida, Tetraphidopsida, and Cycadopsida.

Additionally, the type of eukaryote macro- or micro- was added to the annotation process done by the volunteers.

To add an extra layer of corroboration to our curation process, we assigned microbial species to, at least, three different randomized volunteers. Responses were compared and a majority-voting system was implemented. Answers where there was not a consensus response were labelled as ‘Incongruent’ (e.g. Gram-staining: positive and negative). In questions where multiple choices were allowed (e.g. microbiome source: soil and urban environments), all answers were kept and unified. See Supplemental Fig. 2 for an example.

Cleaning of the data compiled by volunteers was performed using custom scripts to remove typos in scientific names (see GitHub). Additionally, data from previous versions of TMD database [14, 15] were standardized with the newest microbial features and merged into the current dataset. Cleaned data files used to build the database can be found in GitHub (see the Data availability section).

### Data generation with Gemini artificial intelligence

To compare the reliability and expertise of the manual annotation effort versus the speed of AI to annotate microbial characteristics, Gemini was tested using a series of prompts in Google Gemini AI studio v1.5 Flash (https://gemini.google.com/app) using the following link: https://ai.google.dev/aistudio. The prompts were submitted for archaea, bacteria, virus, microfungi, and microalgae. The features that were asked for bacteria and archaea included oxygen use, gram-stain or gram-staining, biofilm formation, spore formation, extremophile, type of extremophile, microbiome, metabolism, and pathogenicity. For virus, genetic material, strand, sense, capsid symmetry, envelope, pathogenicity, and host. For diatoms, type of growth, morphology, biofilm formation, UV resistance, extremophile, type of extremophile, lifestyle, symmetry, toxicity, antagonistic activity, and symbiosis.

The prompts submitted included the following statements: ‘Access PubMed and filter research papers in the field of microbiology that describe [domain] and mention any of the following characteristics: [feature 1, feature 2, feature 3, feature 4, etc.]’. Additionally, some facts were added to the prompts to guide Gemini while compiling the microbial data. The facts included ‘Archaea are not bacteria’ and ‘Gram-staining can be positive or negative’. To view the full list, please refer to Supplementary information. The prompt for each microbial group was submitted at least three times to get as many species entries as possible. No changes between resubmission of the prompts were made. After the third trial ~70% of the species names were duplicated, and the search effort was stopped.

Additionally, the free version of ChatGPT (version GPT-4o) by OpenAI [16] was tested to perform annotations with the prompts previously described for Gemini AI. However, ChatGPT was unable to directly access PubMed or other external databases for research papers. Since the purpose of the comparisons with TMD is to provide free annotations, we did not try the ChatGPT PLUS version.

### External datasets

To demonstrate the utility of TMD database, four publicly available whole genome sequencing (WGS) datasets were downloaded: The Human Microbiome Project (HMP) [17], MetaSUB [18], TARA Oceans [19], and Sponge Microbiome Project (SMP) [20, 21]. For HMP, 19 randomized WGS samples were selected, including male and females. These samples comprised different body sites such as gingiva, gastrointestinal tract, right retroauricular crease, throat, and vagina (Supplementary Table 2). From the MetaSUB project, 24 WGS samples were downloaded. These included nine cities in nine countries with at least two different samples per city. Samples were selected semi-randomly to comprise beach and no-beach cities (i.e. beach city = Barcelona, Spain vs. no beach city = Berlin, Germany). From the TARA Oceans dataset, 10 samples from the Polar circle region were randomly downloaded. Lastly, from the SMP, two randomized samples from three sponge species from the genus *Ircinia* were selected (*n* = 6 total). Accession numbers for all samples can be found in Supplementary Table 2.

Raw FASTQ files from all datasets were downloaded and processed with the same computational pipeline, including taxonomic classification with Kraken2 with a custom microbial genome database that include all microbial genomes in RefSeq. Statistical analysis and comparisons with the TMD database were performed with custom R scripts, which can be found in GitHub: https://github.com/mariaasierra/themicrobedirectory (see the Data availability section).

**Table 1.** Comparison of the Microbe Directory database and other publicly available microbial databases. Some of the characteristics that are included in TMD are individually found in some databases. [8, 9, 22–40]

			Reference publication	Database publication year	the microbe directory																		
Database	Description	Link database			Bacteria Archaea	Fungi	Algae	Virus	Gram- Stain (Bacteria)	Microbiome	Metabolism	Pathogen	Host	Human body site	Spore forming	Oxygen use	Biofilm forming	Extrem-ophile	Anti-microbial Resistance	Genetic material (Virus)	Strand (Virus)	Envelop (Virus)	Capsid (Virus)
**BACMAP Genome Atlas**	BacMap is an interactive visual database containing hundreds of fully labeled, zoomable, and searchable maps of bacterial genomes	http://bacmap.wishartlab.com	[22]	2005	×				×	×	×	×	×										
**ATCC**	American Type Culture Collection collects, stores, and distributes standard reference microorganisms	https://www.atcc.org/About	[23]	1927	×				×		×	×						×	×	×			
**VFDB**	Virulence factors of pathogenic bacteria	http://www.mgc.ac.cn/cgi-bin/VFs/v5/main.cgi	[24]	2004	×				×		×	×	×			×							
**BV-BRC**	Bacterial and Viral Bioinformatics Resource Center for bacterial and viral infectious diseases	https://www.bv-brc.org	[25]	2023	×			×	×			×	×						×		×		
**PHIDIAS**	The Pathogen-Host Interaction Data Integration and Analysis System (PHIDIAS) for pathogens with high priority in public health and biological defense.	https://phidias.us/introduction.php	[26]	2007	×				×			×			×	×				×	×	×	
**eHOMD**	Human Oral Microbiome Database provides a comprehensive curated information o­n the bacterial species present in the human aerodigestive tract	http://www.homd.org	[27]	2010	×				×	×		×		×	×								
**BacDive**	The Bacterial Diversity Metadatabase	https://bacdive.dsmz.de/advsearch	[9]	2013	×				×	×	×	×	×	×	×	×		×	×				
**GOLD**	Genomes Online Database	https://gold.jgi.doe.gov/organisms	[8]	1997	×	×	×	×	×		×				×	×							
**GBIF**	Global Biodiversity Information Facility. Free and open access to biodiversity data	https://www.gbif.org/species/search	[28]	2001	×	×	×	×		×													
**ThermoBase**	A Centralized Database of Thermophiles and Hyperthermophiles	https://exprotdb.com/organisms_browse.php	[29]	2022	×	×				×	×					×		×					
**HaloDom**	Halophilic organisms across all domains	http://halodom.bio.auth.gr/?view=all_data	[30]	2017	×	×	×											×					
**BaAMPs**	Database dedicated to antimicrobial peptides (AMPs) specifically tested against microbial biofilms.	http://www.baamps.it	[31]	2015	×												×						
**U.S National Fungus Collections**	USDA Agricultural Research Service	https://fungi.ars.usda.gov	[32]	1890		×							×										
**Microfungi Collection Consortium**	The Microfungi Collections Consortium (MiCC) is a collaborative effort to digitize specimen label data from North American microfungi	https://www.microfungi.org	NA	2014		×				×													
**Mycology Collection Portal**	MyCoPortal is data derived from a network of universities, botanical gardens, museums, and agencies that provide taxonomic, environmental, and specimen-based information	https://www.mycoportal.org/	[33]	2012		×				×													
**EmsemblFungi**	Ensembl Fungi is a browser for fungal genomes.	https://fungi.ensembl.org	[34]	2002		×						×	×		×								
**UNITE**	Database and sequence management environment centered on the eukaryotic nuclear ribosomal ITS region	https://unite.ut.ee/index.php	[35]	2003		×	×						×										
**FungalTraits**	Traits database of fungi and fungus-like stramenopiles	https://docs.google.com/spreadsheets/d/1cxImJWMYVTr6uIQXcTLwK1YNNzQvKJJifzzNpKCM6O0/edit?usp=sharing	[36]	2019		×			×			×											
**ViruSITE**	Integrated database for viral genomics	http://www.virusite.org/index.php?nav=browse	[37]	2016				×					×							×	×		
**IRAM**	Virus Capsids Database & Analysis Resources	NA	[38]	2019				×															×
**VIPERdb**	Virus Particle Explorer: a web interface for exploring virus structures	https://viperdb.org	[39]	2001				×					×							×	×		×
**Virus-Host DB**	Organizes data about the relationships between viruses and their hosts	https://www.genome.jp/virushostdb/	[40]	2016				×				×	×							×	×		
**Diatoms.org**	Documents diversity of diatoms in North America, providing information on diatom identification, ecology and distribution	https://diatoms.org/	[10]	2022			×			×													

## Results and discussion

### A centralized and accessible source of information

There are several online biological databases for microbial information (Table 1); however, each of them has limitations. For instance, most databases are dedicated exclusively to a specific group of organisms or characteristics (e.g. a database solely for bacteria or one for human pathogens) [41]. Consequently, there is not a centralized repository for downloading information on microbes from different domains that share a specific characteristic (e.g. all human pathogenic bacteria, fungi, and viruses) or for obtaining data on multiple characteristics across different groups of microorganisms (e.g. extremophile microbes in water microbiomes). Additionally, many databases require coding expertise to retrieve information for more than one organism (e.g. through Application Programming Interface (API) download) [42, 43]. While some databases offer tutorials on how to download this data, these can be time-consuming and may require the installation of additional packages. Furthermore, although most databases are peer-reviewed and published, their online resources may not remain active or maintained, rendering the database temporary and potentially unusable for future research [44, 45]. Lastly, current databases do not facilitate easy retrieval of custom lists of microbial species. This is especially critical when analysing metagenomic results, which can generate lists of hundreds or thousands of species, making manual investigation of each species practically impossible [46]. In contrast, TMD provides a centralized and comprehensive database that is easily accessible, searchable, and downloadable. This resource offers significant support to researchers by delivering a user-friendly platform for the broadscale understanding of microbial species, facilitating the generation of ecological hypotheses, and driving correlations.

**Figure 1.**
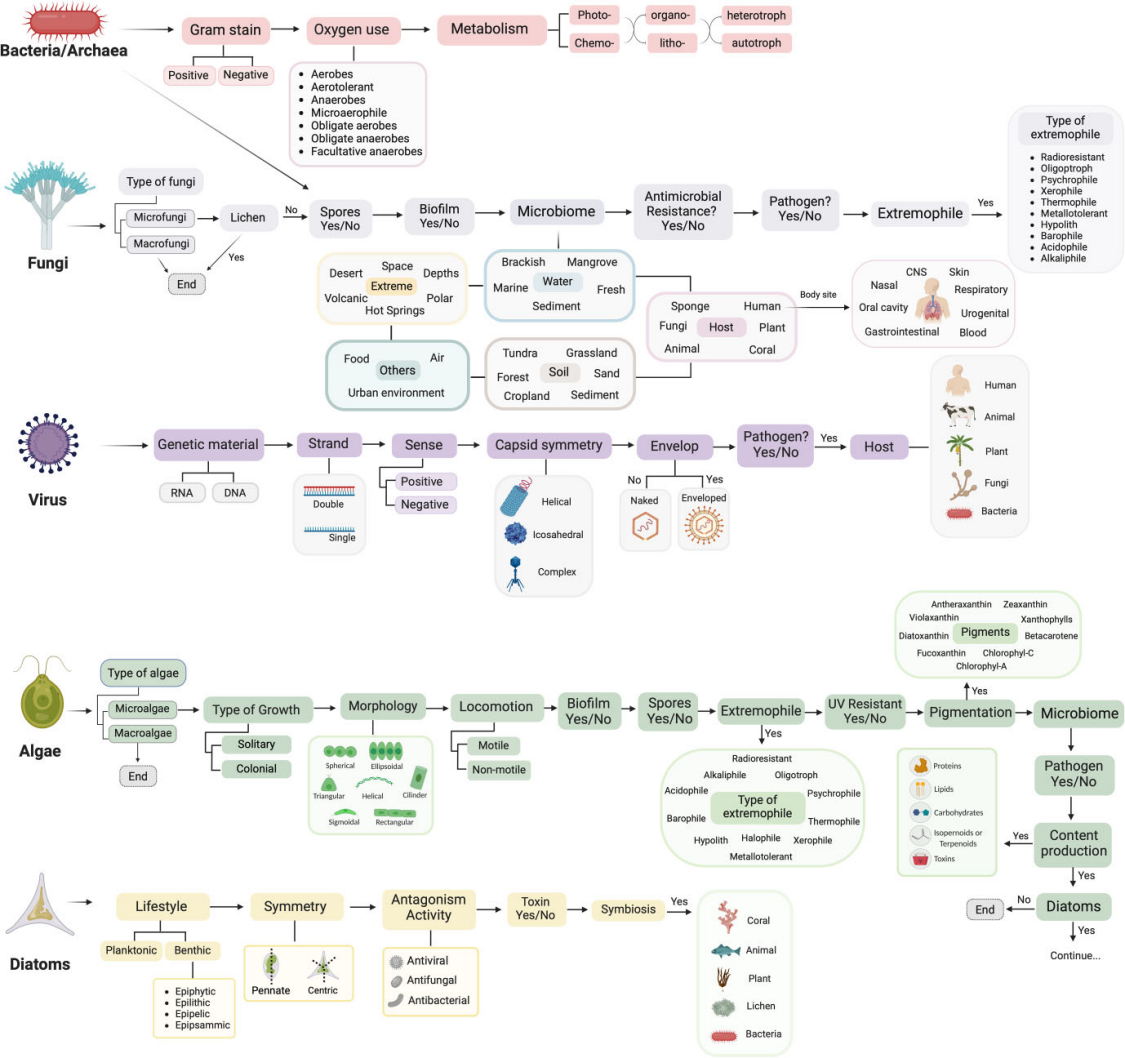
Structure of The Microbe Directory database. The information collected has been targeted by microbial domains. Data collected followed the schema shown.

To compile and centralize the most relevant information on microbial species, TMD organizes its data by domains (Fig. 1). Each domain is annotated based on its phenotype, such as Gram-staining for bacteria or genome type for viruses, and its ecological features, such as the source of microbiome, host, and extremophiles. Additionally, to address relevance in ecology or biotechnology, algae have been further divided into diatoms and other microalgae [47, 48], and a question regarding the multicellular or unicellular identity of fungi and algae has been added. This last addition recognizes the complexity arising from multicellularity spanning across various lineages [13, 49] and provides an initial list of only-macro- or only-microorganisms for other researchers.

**Figure 2.**
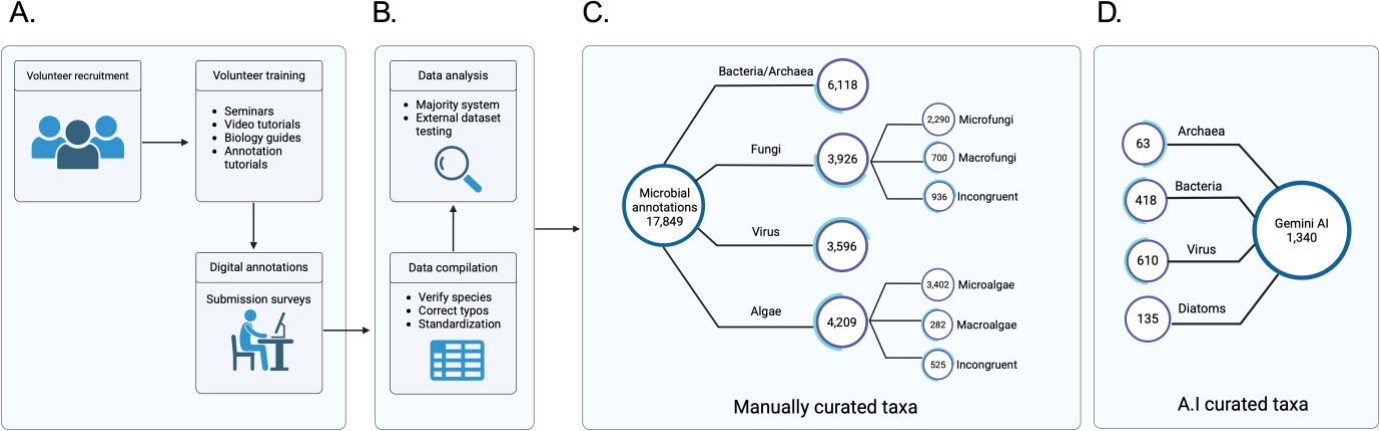
Diagram of The Microbe Directory database. (A) Volunteer recruitment, training, and data annotation. (B) Processing of the data manually collected. (C) Database construction using curated data. (D) Comparison and inclusion of AI-collected data.

TMD database is composed of 17,849 curated microbial species from all domains of life, bacteria, archaea, virus and eukarya, including microfungi, and microalgae (Fig. 2). The compilation process is described in the Methods section. Briefly, a list of species names from bacteria, archaea, virus, fungi, and algae were downloaded from NCBI taxonomy and cleaned from redundant names. These species were assigned randomly in triplicates to almost 400 student volunteers. The volunteers were trained and provided with guidance on how to annotate characteristics of the microorganisms assigned before submitting the information into a predesigned online survey. After submitting, the information was compiled and cleaned for typo errors. Answers to questions with a unique answer were submitted to a voting system to identify the most probable answer (Supplementary Fig. 2). For instance, if the same bacterial species was assigned to three different volunteers, and one volunteer submitted it as a Gram-staining negative, but two others submitted it as a Gram-staining positive, the species ultimately was classified as Gram-staining positive based on a majority vote. In cases where the species was assigned only to an even number of volunteers (two or four volunteers), and there was not a consensus vote, the answers were classified as *Incongruent*. This provides an additional corroboration step and allows us to go back to those problematic species in future versions of the database. Additionally, data generated through Gemini AI (see the Methods section) were compared and merged into the database for further analysis.

### A user-friendly database interface easy to navigate and open access

The Microbe Directory website dashboard was created using ShinyApps by R. It has a three-pronged use case. Firstly, for exploratory analysis, manually curated datasets are available for search using the ‘Choose a Dataset’ tab. Using this tab, users can filter for particular terms in each column of the database and download the resulting subset as a CSV file. The datasets include an all-encompassing microbe directory (TMD) and subsets specific to bacteria, archaea, algae, fungi, and viruses. The dashboard also includes a ‘Batch Query’ tab, which allows users to submit a list of scientific names of microbes of interest. If included in TMD, annotations corresponding to the query microbes are returned as a table that can be downloaded in CSV format. Furthermore, to help with exploratory analysis, an ‘Exploration’ tab is available. Users can either carry over the results from the ‘Batch Query’ tab or choose one of the pre-curated datasets (i.e. TMD, Bacteria, Archaea, Algae, Fungi, Virus). Users can explore the dataset they choose by selecting an annotation column (e.g. microbiome, extremophile_type, biofilm_forming), which includes a description of the term. After selecting an annotation column to visualize, an interactive plot is rendered in which either the entire set or an intersection between multiple sets can be chosen by clicking on certain highlighted regions on the plot. Through this action, a subset of the dataset is generated based on which set was clicked on. Users can download this subset or the excluded data of the chosen subset using the respective download buttons. Users can also filter within the generated subset table and download the complement dataset.

### How to use The Microbe Directory database to aid in metagenomic analyses

The purpose of TMD is not to conduct taxonomic classification during microbiome analysis. Rather, TMD serves as an information database of species, allowing for cross-referencing species names obtained from any taxonomic analysis to aid in the characterization of the taxa. To test the utility of TMD, taxonomic results from four metagenomic datasets were used (Supplementary Table 2).

**Figure 3.**
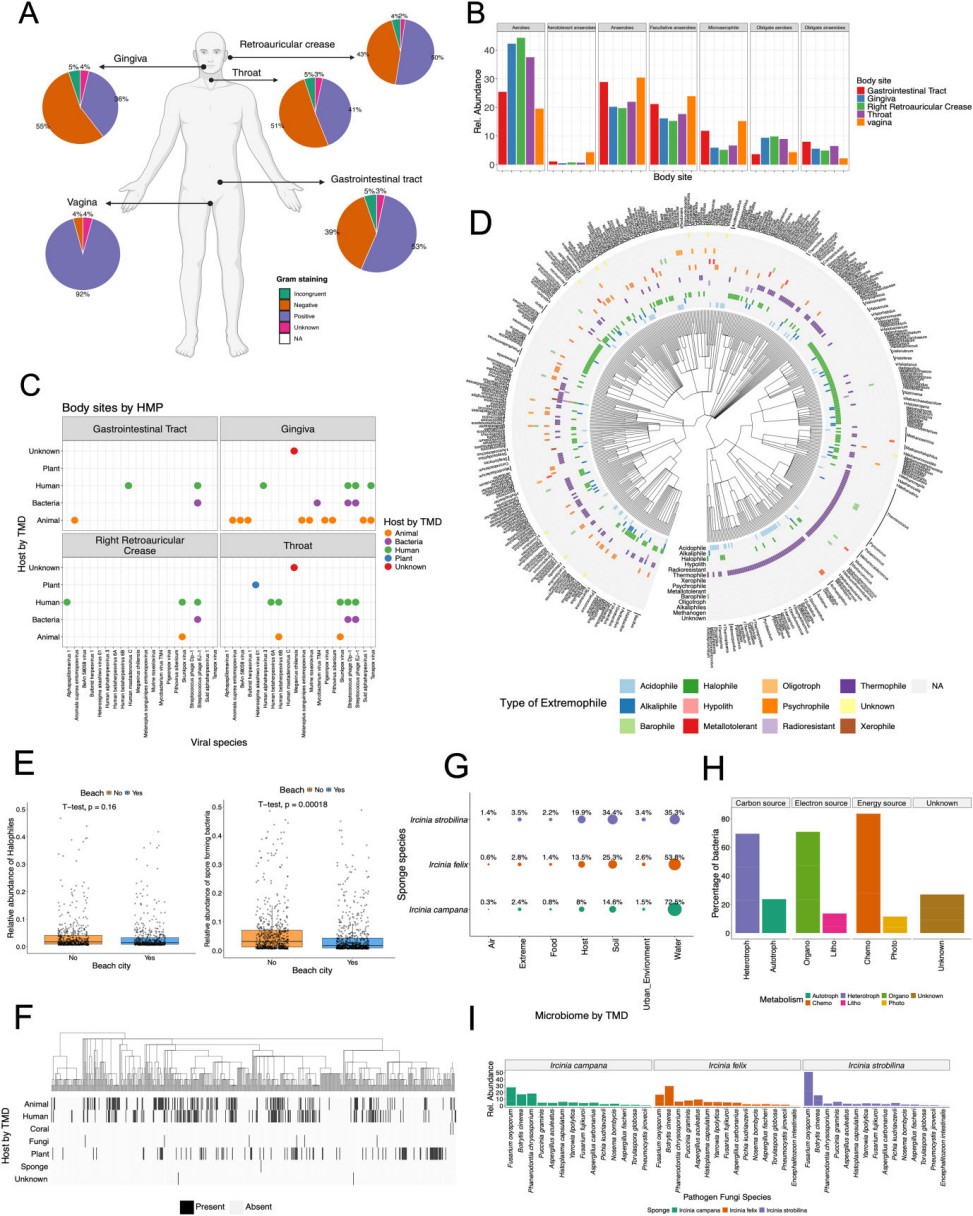
Example uses of The Microbe Directory (TMD) with external datasets. (A–C) Whole genome sequencing (WGS) data from the Human Microbiome Project (HMP). (A) Percentage of bacteria by Gram-staining in each body site. (B) Relative abundance of bacteria by their oxygen requirements across body sites. (C) Viral species in each body site and their potential host of origin according to TMD. (E–F) WGS data from MetaSUB project. (E) Correlations of relative abundance of halophile (salt-loving microorganism) and spore-forming bacteria in cities close to the beach. (F) Heatmap of bacteria and their potential host of origin according to TMD. (G–H) WGS from Sponge Microbiome Project. (G) Percentage of bacteria from different microbiomes according to TMD in the three sponge species. (H) Percentage of bacteria and their different types of metabolism present in the sponge microbiome. (I) Relative abundance of pathogenic fungi present in all three sponge species.

Using data from the HMP [49], three example questions were investigated: What are the ratios of Gram-staining positive and negative bacteria in each body site, what are the oxygen requirements of bacteria in these sites, and what are the potential sources of viruses present in each body site? By employing TMD to annotate the metagenomic results of HMP data, some physiological trends were found (Fig. 3A). For example, a similar proportion of Gram-negative and Gram-positive bacteria was observed in the gingiva, throat, gastrointestinal tract, and retroauricular crease, although Gram-positive bacteria appeared to be distinctively prevalent in vagina. This result is consistent with metagenomic studies of the vagina microbiome, which have shown that in a healthy state, the Gram-positive bacteria *Lactobacillus* sp. is one of the most dominating taxa [50]. Similarly, bacteria from vagina, as well as the gastrointestinal tract, differed from other body sites on their bacteria oxygen requirements, with higher abundances of anaerobes, facultative anaerobes, and microaerophiles (Fig. 3B). The genus *Lactobacillus*, in fact, is also classified as an anaerobe, and previous research supports the higher abundance of anaerobes and microaerophiles in the vagina microbiota [50, 51]. Most of the species present in the HMP dataset are suspected to be part of the human commensal microbiome, including viruses such as herpesviruses, known to be highly widespread in the human population [52]. However, some viruses present in the HMP have other non-human origins, such as animals or plants (Fig. 3C). The highest heterogeneity of virus hosts corresponds to the gingiva and throat, which provides support to the notion that exogenous elements and microbes might be transported to the body through food, water, or air [53].

Another dataset used was the TARA Oceans metagenomes from the polar circle region [18]. Leveraging TMD extremophiles annotations, TMD provided valuable, and possibly novel insights into the presence and diversity of extreme-living microbes (known as extremophiles), in TARA metagenomes. By comparing the taxonomic names retrieved from TARA Oceans microbiome, with the extremophile lists in TMD, 631 taxa were catalogued as extremophiles in TARA, and revealed the presence of 11 distinct types of extremophiles (Fig. 3D), with certain species adapted to more than one form of environmental stress such as tolerant to both acidic (acidophile) and saline (halophile) conditions simultaneously. These microorganisms are described as polyextremophiles. Previous reports have shown the presence of psychrophilic and psychrotolerant microbes in polar environments [54], as well as the presence of acidophiles potentially from exposure to penguin guano and halophiles due to pockets of seawater trapped in closed basins [54]. However, TMD analysis showed a high abundance of thermophiles (microbes known to thrive in high temperatures). Thermophiles have been described in Antarctica in thermal springs, fumaroles, hot soils, and hydrothermal vents [55], but not in the polar region where these samples originate. This analysis depicts the possible research gap on high-temperature microbes in polar regions, and the potential novel molecular traits that allow them to thrive in these environments. TMD annotation provides new insights into the ecology of microorganisms that might otherwise go unnoticed.

Additionally, the MetaSUB dataset [17] was used to assess TMD annotations of urban microbiomes and investigate the correlation between salt-loving microbes (halophiles) and their prevalence in coastal cities due to their proximity to high-salinity waters. By employing TMD annotations to classify halophilic microbes in both coastal and non-coastal urban areas, our analysis revealed no statistically significant difference in the abundance of halophiles between coastal and non-coastal cities (Fig. 3E). However, a correlation emerged indicating lower levels of spore-forming bacteria in coastal cities. Few studies have accessed the presence of halophiles in city beaches [56, 57]. But no studies have performed a systematic comparison between non-coastal and coastal cities. Additionally, the higher presence of spore-forming microbes in non-coastal cities could be correlated to the season temperature, as previous research has shown an increase in the spore-forming bacteria *Bacillus* after heatwaves [58]. The analysis with TMD shows that more research into the microbial ecology of cities is needed.

Next, TMD analysis revealed that as expected due to the nature of the MetaSUB dataset, most species found in MetaSUB metagenomes originate from humans. However, there were some additional microbes originating from animals and plants (Fig. 3F). These results are consistent with previous studies describing the correlation between the human skin microbiome and the environment [59], as well as the influences of lifestyle and urban or rural settings on the microbiota of skin, saliva, and gut [60, 61].

An additional analysis using the sponge microbiome dataset taxonomic result [19, 20] and the TMD annotations revealed that the majority of microbial species in sponge metagenomes could be classified as microorganisms coming from water microbiomes (Fig. 3G). A smaller fraction originated from soil or other host microbiomes. This result differs from the original dataset publication where authors showed that, using 16S metabarcoding, microbes in the sponges differ from those in the seawater, with a small subset taxa overlap [19]. This might be because TMD depends heavily on previously published taxa, where microbiomes of hosts such as sponges and corals are underrepresented compared to water or soil microbiomes.

Additionally, microbial communities in sponges exhibited diverse metabolic capabilities according to TMD annotations, with chemo-organo-heterotrophs predominating (Fig. 3H). This observation aligns with previous findings examining metabolic pathways across various *Ircinia* sponge microbiomes, where genes associated with nitrogen, sulphur, and carbon metabolism were highlighted [20]. Finally, TMD helped to unveil the presence of pathogenic fungi in all three *Ircinia* sponge species (Fig. 3I). Although the abundance of these fungal species differed among the sponge species, it is noteworthy that marine sponge extracts, particularly from *Ircinia*, have exhibited significant antifungal activity against various pathogens, including *Fusarium oxysporum* [62, 63]. This finding implies a potential interaction or long-term colonization of fungal pathogens within these sponge ecosystems that needs to be further investigated.

When assessing the presence of species from each of the four studies in TMD, we found a consistent overlap of ~25% of taxa. This indicates that TMD is phylogenetically diverse and comprises a wide range of microbial species from various environments and hosts (Supplemental Fig. 3). While a 25% species representation may not be sufficient to draw definitive biological conclusions in a metagenomic study, it can still provide valuable insights into the biology and ecology of microbiomes. We anticipate that future versions of TMD will include a greater overlap of species.

### Artificial intelligence versus domain of knowledge

**Figure 4.**
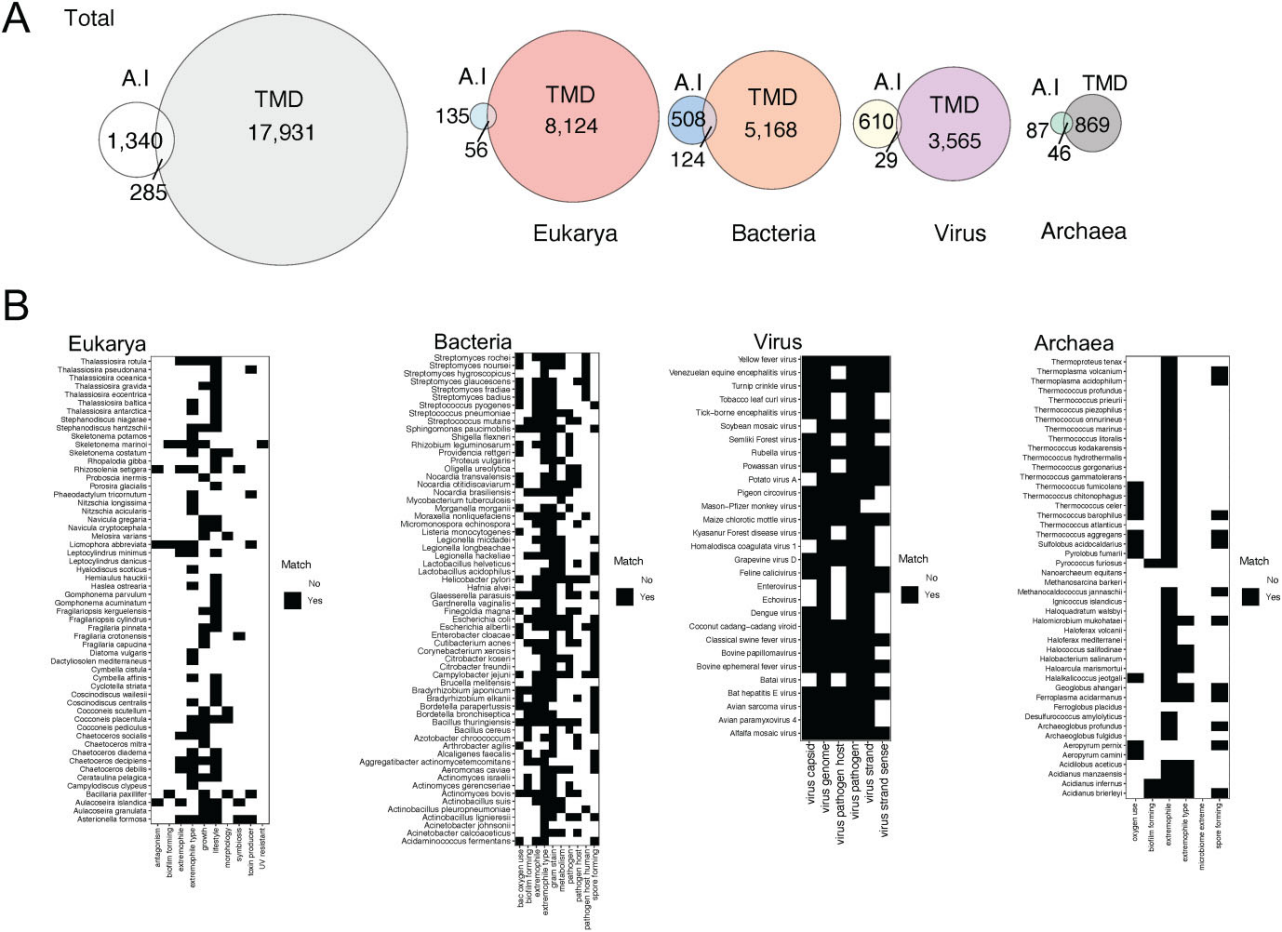
Comparison of Gemini AI and TMD annotations. (A) Overlapped and unique number of taxa in the total database and in each domain by each annotation approach (Gemini AI or TMD). (B) Matrix of annotations by AI compared with TMD in the overlapped taxa. Characteristics that match are depicted in black and discrepancies in white.

Utilizing microbial annotations generated by Gemini AI by Google (See the Methods section), using search prompts, our search yielded 1340 taxa spanning bacteria, archaea, virus, and eukarya. Among these, 285 taxa overlapped with TMD annotations (Fig. 4A), with Archaea exhibiting the highest ratio of overlap (52%) and viruses the lowest (4.7%). Despite this low ratio, virus species showed the highest level of matching results with TMD annotations. Phenotypic traits like type of genome or genome strand demonstrated the highest concordance, whereas ecological features such as virus host exhibited greater disparity (Fig. 4B). These disparities in ecological features stemmed from instances where the AI provided ‘No’ responses for traits categorized as ‘Unknown’ data by TMD. Antagonistic activity, UV resistance, and biofilm formation are among the characteristics catalogued as ‘No’ by AI due to insufficient literature, whereas TMD volunteers recognized the gaps in research and literature and catalogued them as unknown.

Recently, AI tools have been developed to help with the laborious literature review process [64, 65]; however they require input libraries of articles to be screened by the AI tools. Given that the annotations by TMD are aimed for thousands of taxa, and multiple microbial characteristics, providing an input library to train AI models would be challenging. Hence, Gemini AI emerged as an alternative method, free to date, for gathering microbial data through prompts. Nevertheless, despite repeated submissions, Gemini consistently provided annotations for the same taxa, thereby restricting the breadth of accessible species through this approach. The limited accessibility of open-access research articles and Gemini's utilization of archives like PubMed might contribute to the limited number of AI microbial annotations, something that can also be a limiting factor for manual annotations if no institutional access is available. Unfortunately, during the revision process of this work, Gemini AI changed its model and no longer allowed access to external sources such as PubMed. Despite these limitations, freely available tools like Gemini could be used to guide manual annotations ultimately supporting the domain of knowledge.

### The Microbe Directory builds opportunities between students and research

TMD database is the result of collaborative efforts involving hundreds of students who contributed through manual annotation work. Notably, TMD has offered invaluable learning opportunities amidst the challenges of the COVID-19 pandemic, enabling students to delve into microbiology, scientific literature analysis, and research methodologies. Originally conceived as an in-person project, TMD seamlessly transitioned into a remote collaboration platform, providing continuous training from 2019 to 2022. Faced with restrictions on in-person research activities during the pandemic, many students opted to dedicate additional hours weekly to delve deeper into microbial research, utilizing TMD as a platform for internships and earning research credits. TMD evolved into a foundational project for open research, facilitating remote scientific collaboration and engaging a larger number of students in scientific endeavours.

## Conclusion

TMD provides a centralized and curated source of biological and ecological information of microbes from all realms, providing an understanding to the exponential growing dataset generated by sequencing studies. Using external datasets, we show the utility of TMD to understand metagenomic data and to unveil novel trends. However, as any biological database, TMD is far from complete, and authors should interpret results accordingly. As we continue to expand TMD, we hope that in the future more species can be annotated and biological interpretations are more robust.

Moreover, the TMD interface presents a user-friendly visualization of the database, catering to a diverse audience including researchers and students. Additionally, by juxtaposing TMD results with annotations derived from AI, we highlight the limitations that AI encounters in systematic literature review processes compared to the manual curation process of microbial data. Lastly, TMD bridges the gap between research and students by actively involving students from various backgrounds in data collection and analysis.

## Supplementary Material

baaf060_Supplemental_Files

## Data Availability

Data from The Microbe Directory can be found in the database interface: www.themicrobedirectory.com. Raw data from The Microbe Directory domains can be found in GitHub, along with scripts to create main figures, curated lists of species, and Gemini data: https://github.com/mariaasierra/themicrobedirectory. All metagenomic data used for technical validation can be found directly on the studies cited, or using the accession numbers in Supplementary Table 2, and abundance tables in the GitHub. Annotation tutorials can be found in YouTube (https://www.youtube.com/@themicrobedirectory9883) and microbiology guides on Coda (https://coda.io/d/Microbiology-Guides_dI4sBF6pufK/The-Microbe-Directory_supfr#_luCYc).
