# Supplementary material for: The Microbe Directory: a centralized database for biological interpretation of microbiome data
Source: Database (Oxford). 2025 Sep 24;2025:baaf060. doi: 10.1093/database/baaf060 (PMC12462379; doi:10.1093/database/baaf060)
Supplement: baaf060_Supplemental_Files [file baaf060_supplemental_files.zip › Supplementary Figure 1.pdf]

# The Microbe Directory: Data Entry Form (Volunteers Winter - Spring 2021)

## Welcome to the Microbe Directory

For further information please follow the link:

https://github.com/dcdanko/MD2/blob/master/README.md

*Scientific name

*Please give genus species name Enter as assigned.*

*Specify Domain

- ( ) Bacteria/Archaea
- ( ) Eukarya
- ( ) Virus

Please answer each question in sequential order. Your response to each question will determine which questions will appear next.

*If you are unsure how to proceed, please refer to the website link above or email **themicrobedirectory@gmail.com***

*Enter your full name

*Please enter your name the same on every entry.*

✔ Validate

## Bacteria / Archaea

*Oxygen use

- [ ] Aerobes
- [ ] Obligate aerobes
- [ ] Anaerobes
- [ ] Obligate anaerobes
- [ ] Facultative anaerobes
- [ ] Aerotolerant anaerobes
- [ ] Microaerophile
- [ ] Unknown
- [ ] N/A

*Gram stain

- ( ) Negative
- ( ) Positive
- ( ) Unknown
- ( ) N/A

*Is it an Extremophile?

- ( ) Yes
- ( ) No
- ( ) Unknown
- ( ) N/A

*Spore forming

- ( ) Yes
- ( ) No
- ( ) Unknown
- ( ) N/A

*Microbiome

- [ ] Host
- [ ] Soil
- [ ] Water
- [ ] Extreme
- [ ] Urban Environment
- [ ] Food
- [ ] Air
- [ ] Unknown
- [ ] N/A

*Pathogen

- ( ) Yes
- ( ) No
- ( ) Unknown
- ( ) N/A

*Biofilm forming

- ( ) Yes
- ( ) No
- ( ) Unknown
- ( ) N/A

*Antimicrobial resistance

- ( ) Yes
- ( ) No
- ( ) Unknown
- ( ) N/A

*Type of metabolism

- [ ] Photo
- [ ] Chemo
- [ ] Organo
- [ ] Litho
- [ ] Heterotroph
- [ ] Autotroph
- [ ] Unknown
- [ ] N/A

Enter the reference(s) you used to find the information above. Please use the site ZoteroBib in order to standardize your entries:

## Eukarya

What type of Eukarya?

- (•) Fungi
- ( ) Algae

*What type of Fungi?

- ( ) Microfungi
- ( ) Macrofungi

*Is it a lichen?

- ( ) Yes
- ( ) No
- ( ) Unknown
- ( ) N/A

*What type of Fungi?

- (•) Microfungi
- ( ) Macrofungi

*Is it a lichen?

- ( ) Yes
- ( ) No
- ( ) Unknown
- ( ) N/A

*Is it an Extremophile?

- ( ) Yes
- ( ) No
- ( ) Unknown
- ( ) N/A

*Spore forming

- ( ) Yes
- ( ) No
- ( ) Unknown
- ( ) N/A

*Microbiome

- [ ] Host
- [ ] Extreme
- [ ] Soil
- [ ] Water
- [ ] Urban Environment
- [ ] Food
- [ ] Air
- [ ] Unknown
- [ ] N/A

*Is it a pathogen?

- (•) Yes
- ( ) No
- ( ) Unknown
- ( ) N/A

*Host

*Pathogen*

- [ ] Plant
- [ ] Animal
- [ ] Fungi
- [x] Human
- [ ] Unknown
- [ ] N/A

*Location in body

*Human host*

- [ ] Blood
- [ ] Gastrointestinal tract
- [ ] Skin
- [ ] Oral Cavity
- [ ] Urogenital tract
- [ ] Nasal Passage
- [ ] Respiratory Tract
- [ ] CNS
- [ ] Unknown
- [ ] N/A

*Does it produce pigmentation?

- ( ) Yes
- ( ) No
- ( ) Unknown
- ( ) N/A

*Does it have mobility?

- ( ) Motile
- ( ) Non-motile
- ( ) Unknown
- ( ) N/A

*Does it form a biofilm?

- ( ) Yes
- ( ) No
- ( ) Unknown
- ( ) N/A

*Is it an Extremophile?

- ( ) Yes
- ( ) No
- ( ) Unknown
- ( ) N/A

*Type of reproduction

- ( ) Sexual
- ( ) Asexual
- ( ) Sexual and Asexual
- ( ) Unknown
- ( ) N/A

*Does it form spores?

- ( ) Yes
- ( ) No
- ( ) Unknown
- ( ) N/A

*Does it form symbiosis?

- ( ) Yes
- ( ) No
- ( ) Unknown
- ( ) N/A

*Is it a pathogen?

- ( ) Yes
- ( ) No
- ( ) Unknown
- ( ) N/A

*Content production

*Biotechnology, medical, environmental*

- ( ) Yes
- ( ) No
- ( ) Unknown
- ( ) N/A

*Resistant to UV

*Ultraviolet radiation*

- ( ) Yes
- ( ) No
- ( ) Unknown
- ( ) N/A

## Virus

*Genetic material

- ( ) RNA
- ( ) DNA
- ( ) Unknown
- ( ) N/A

*Strand

- ( ) ss
- ( ) ds
- ( ) Unknown
- ( ) N/A

*Sense

*Sense/Polarity*

- ( ) Positive-strand
- ( ) Negative-strand
- ( ) Unknown
- ( ) N/A

*Capsid

- ( ) Enveloped
- ( ) Naked
- ( ) Unknown
- ( ) N/A

*Pathogen

- ( ) Yes
- ( ) No
- ( ) Unknown
- ( ) N/A
