# Supplementary material for: The Microbe Directory: a centralized database for biological interpretation of microbiome data
Source: Database (Oxford). 2025 Sep 24;2025:baaf060. doi: 10.1093/database/baaf060 (PMC12462379; doi:10.1093/database/baaf060)
Supplement: baaf060_Supplemental_Files [file baaf060_supplemental_files.zip › Supplementary information.docx]

**Supplementary information:**

**List of guides for Gemini prompts.**

- “Archaea are not bacteria”
- “Gram- staining can be positive or negative.”
- “Oxygen use can be aerobe or aerobic, aerotolerant, anaerobe or anaerobic, microaerophile, obligate anaerobe, obligate aerobe, or facultative anaerobe.”
- “Biofilm formation is the capacity of a microbe to form biofilms. Should be a binary answer: Yes or No.”
- “Spore formation is when the microbe forms spores to resist harsh conditions or go dormant. Should be a binary answer: Yes or No.”
- “An extremophile is a microbe that can survive and thrive in extreme environments.”
- “Extremophiles can be multiple types. For example: Psychrophile, Thermophile, Acidophile, etc.”
- “The same microbe species can be multiple extremophile types at the same time.”
- “Microbiome refers to where the microbe is found, for example hot-springs, ocean, soil, etc.”
- “Bacteria/Archaea can be heterotroph or autotroph depending on the carbon source. They can also be organotroph or lithotroph depending on the electron source. They can also be chemotroph or phototroph depending on the energy source.”
- “Pathogens cause disease in the host. The host can be humans, animals, plants, fungi, corals. If the host is human, then tell what body site it infects. Body sites can be CNS, gastrointestinal, blood, skin, respiratory, urogenital, nasal, oral cavity.”
- “A microbe is not considered a pathogen if they only cause disease in immunosuppressed patients.”
- “Diatoms are algae, but not all algae are diatoms.”
- “The type of growth of diatoms can be solitary or colonial.”
- “Morphology of diatoms can be many, including circular, triangular, cylinder, etc.”
- “UV protection indicates if the diatom/algae are resistant to UV light.”
- “Lifestyle of diatom/algae can be planktonic or benthic.”
- “Symmetry of diatom can be pennate or centric.”
- “Toxicity indicates if the diatom produces toxins.”
- “Antagonistic activity indicates if the diatom produces any compound that can antagonize the growth of any other organism such as antagonize viruses called antiviral, antagonize fungus called anti-fungal, or antagonize bacteria called antibacterial.”
- “Symbiosis is when a diatom has a symbiotic relationship with another organism, such as corals, animals, plants, sponges, lichens, or bacteria. Symbiosis answer should be binary: Yes or No.”
- “Symbiosis host indicates the organism with who the diatom forms the symbiosis.”
- “For virus genetic material can be RNA or DNA.”
- “Strand refers to the type of strand RNA or DNA. It can be double or single stranded. It can be summarized as dsDNA, ssDNA, ssRNA, dsRNA.”
- “Sense references the sense of the genetic strand. It can be positive or negative.”
- “Virus capsid refers to symmetry and can be helical, Icosahedral, or complex.”
- “Envelop refers to the outermost layer of the virus. When the virus does not have an envelope, it is called naked. It should be either envelope or naked.”
- “The virus host can be any of the following options: human, animal, plant, fungi, algae, corals.”
