# Supplementary figures and images for: The Microbe Directory: a centralized database for biological interpretation of microbiome data
Source: Database (Oxford). 2025 Sep 24;2025:baaf060. doi: 10.1093/database/baaf060 (PMC12462379; doi:10.1093/database/baaf060)

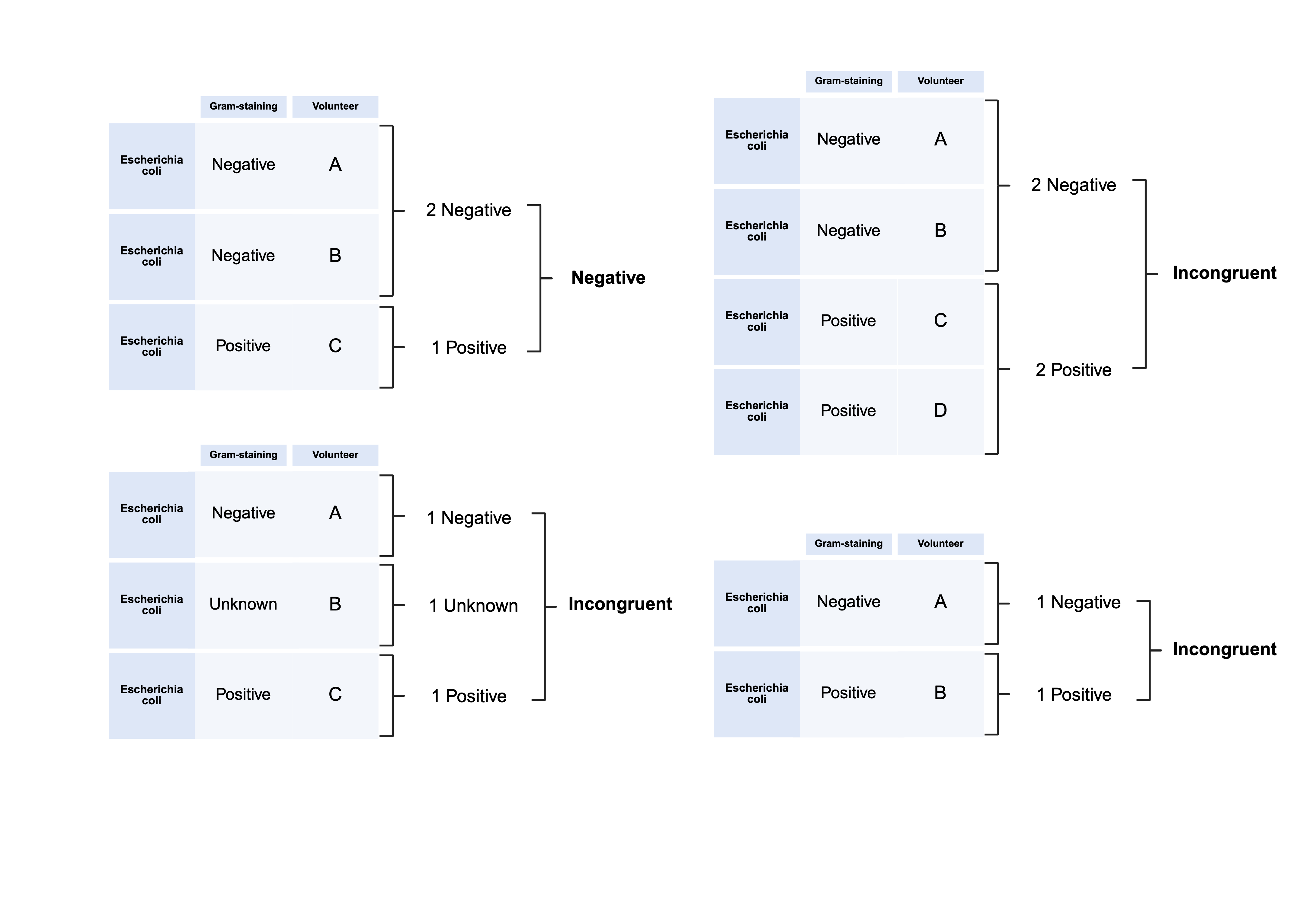

Supplement: baaf060_Supplemental_Files [file baaf060_supplemental_files.zip › Supplementary Figure 2.png]

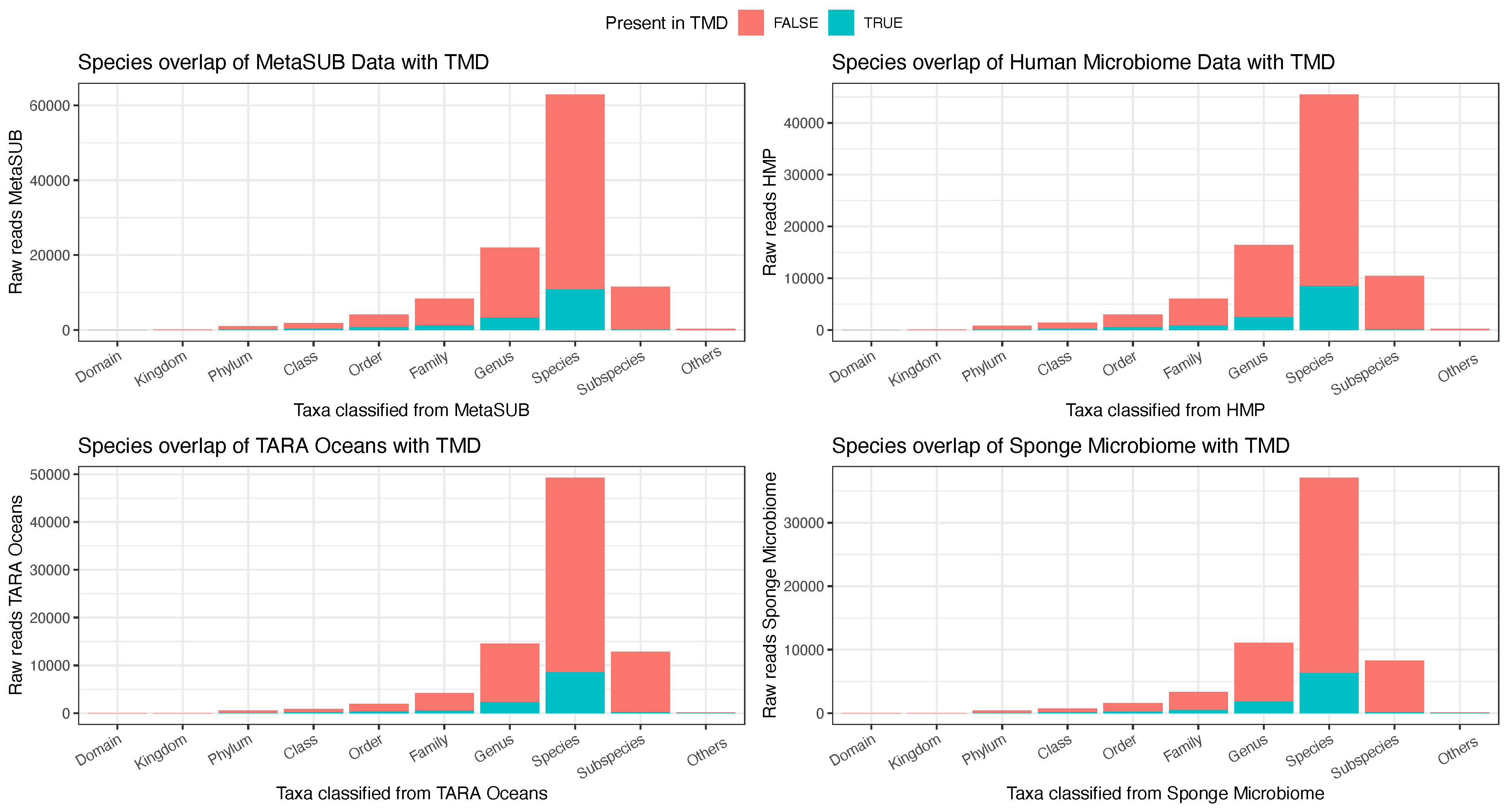

Supplement: baaf060_Supplemental_Files [file baaf060_supplemental_files.zip › Supplementary Figure 3.png]
